# A CT-based interpretable deep learning signature for predicting PD-L1 expression in bladder cancer: a two-center study

**DOI:** 10.1186/s40644-025-00849-1

**Published:** 2025-03-10

**Authors:** Xiaomeng Han, Jing Guan, Li Guo, Qiyan Jiao, Kexin Wang, Feng Hou, Shunli Liu, Shifeng Yang, Chencui Huang, Wenbin Cong, Hexiang Wang

**Affiliations:** 1 Department of Radiology, The Affiliated Hospital of Qingdao University, 16 Jiangsu Road, Qingdao, Shandong 266003 China; 2 Department of Radiology, The Fourth Hospital of Shijiazhuang, Shijiazhuang, Hebei 050000 China; 3 College of Computer Science and Technology, Qingdao University, Qingdao, Shandong 266071 China; 4 Qingdao Medical College, Qingdao University, Qingdao, Shandong 266071 China; 5 Department of Pathology, The Affiliated Hospital of Qingdao University, Qingdao, Shandong 266003 China; 6 Department of Radiology, Shandong Provincial Hospital Affiliated to Shandong First Medical University, Jinan, Shandong 250022 China; 7 Department of Research Collaboration, R&d Center, Beijing Deepwise & League of PHD Technology Co., Ltd, Beijing, 100080 China

**Keywords:** Bladder cancer, Computed tomography, Programmed death ligand 1, Deep learning

## Abstract

**Background:**

To construct and assess a deep learning (DL) signature that employs computed tomography imaging to predict the expression status of programmed cell death ligand 1 in patients with bladder cancer (BCa).

**Methods:**

This retrospective study included 190 patients from two hospitals who underwent surgical removal of BCa (training set/external validation set, 127/63). We used convolutional neural network and radiomics machine learning technology to generate prediction models. We then compared the performance of the DL signature with the radiomics machine learning signature and selected the optimal signature to build a nomogram with the clinical model. Finally, the internal forecasting process of the DL signature was explained using Shapley additive explanation technology.

**Results:**

On the external validation set, the DL signature had an area under the curve of 0.857 (95% confidence interval: 0.745–0.932), and demonstrated superior prediction performance in comparison with the other models. SHAP expression images revealed that the prediction of PD-L1 expression status is mainly influenced by the tumor edge region, particularly the area close to the bladder wall.

**Conclusions:**

The DL signature performed well in comparison with other models and proved to be a valuable, dependable, and interpretable tool for predicting programmed cell death ligand 1 expression status in patients with BCa.

**Supplementary Information:**

The online version contains supplementary material available at 10.1186/s40644-025-00849-1.

## Introduction

Bladder cancer (BCa) is one of the most common cancers worldwide, and exhibits a higher prevalence in men than in women [1]. At present, a range of immune checkpoint inhibitors have been acknowledged by the National Comprehensive Cancer Network [2] recommendations for use as second-line treatment for metastatic or advanced uroepithelial carcinoma or for use as first-line treatment for individuals who are not appropriate for cisplatin treatment. Atezolizumab and pembrolizumab, in particular, show significant results in advanced or metastatic BCa.

Immunotherapy has transformed the management of patients with metastatic urothelial malignancies, leading to improvements in survival, progression-free survival, and the durability of responses seen with treatment [3]. In recent years, immunotherapies targeting the Programmed cell death protein 1/programmed cell death ligand 1(PD-L1) axis have made substantial progress [4, 5]. Inhibitors of PD-L1, such as atezolizumab and Durvalumab, have made significant progress in the treatment of bladder cancer [6]. Programmed cell death protein 1 is predominantly expressed in macrophages and lymphocytes, while the ligand for programmed cell death 1 is PD-L1. Research has demonstrated that patients with elevated levels of PD-L1 expression achieve higher rates of objective responses in treatment with immune checkpoint inhibitor immunotherapy in comparison with patients with lower expression [7, 8]. Research indicates that tumors with higher levels of PD-L1 expression are more inclined to be classified as high-grade tumors, and patients with organ-confined disease have lower survival rates and higher rates of postoperative recurrence [9–11]. PD-L1 has become the most valuable biomarker for predicting response to anti-PD-L1 immunotherapy [12].

At present, cystoscopic resection and endoscopic examination remain the standard methods for identifying PD-L1 expression status in BCa. However, partial specimens might not fully represent the whole neoplasm because of dynamic changes and significant intra-tumoral heterogeneity in PD-L1 expression, which can result in inaccurate diagnoses [13, 14]. There is thus a growing necessity to establish a suitable analytical method to predict immune expression outcomes, particularly for individuals who are intolerant of endoscopic examination.

Applying immunohistochemistry staining to invasive biopsy or surgical specimens is the reference standard method for determining the levels of PD-L1 expression [15]. However, immunohistochemistry is time-consuming, and tissue sampling is not always practicable and is linked to elevated expenses, which restricts the utilization of molecular assays reliant on invasive biopsies [16]. Consequently, a new methodology for predicting PD-L1 expression status in clinical applications should be established.

Computed tomography (CT) is frequently employed for the diagnosis and staging of BCa [17]. Radiomics is a quantitative analysis method used to gather information for diagnosing and assessing areas of interest in disease prognosis. Studies [18, 19] have shown that radiomics translates the pathophysiological information of tumors encoded by any form of digital medical imaging into a number of quantitative features that can provide information for clinical decision-making. Cao et al. [20] established a CT-based radiomics model for predicting PD-L1 expression status in BCa. Park et al. [21] built a CT-based radiomics model for predicting the prognosis of programmed cell death 1/PD-L1 immunotherapy for BCa. Deep learning (DL) is a rising image analysis methodology, and numerous research studies have illustrated the significance of CT image-based DL signatures in the diagnosis, treatment, and prognosis of BCa [22–24]. However, the “black box” nature of the DL signature makes it difficult to elucidate the rationale behind specific predictions for patients [25]. Lundberg and Lee introduced the Shapley additive explanation (SHAP) framework to address the “black box” issue and enhance model interpretability [26]. To our knowledge, there is presently no validated interpretable DL signature that uses contrast-enhanced CT to predict PD-L1 expression status in patients with BCa.

The objective of our study was to construct and assess a CT-based DL signature to predict PD-L1 expression status in BCa. At the same time, we incorporated the SHAP framework to visually explain the decision-making process and understand the correlation between PD-L1 expression status in BCa and the DL signature, thereby increasing the model’s dependability for both physicians and patients.

## Methods

### Patient selection

The review boards of all collaborating hospitals approved this retrospective study, which was exempt from the necessity for informed patient consent. This research used data from two healthcare facilities. In accordance with our inclusion and exclusion criteria, we chose patients with BCa who were treated between February 2015 and July 2024. The following inclusion criteria were applied: (a) BCa confirmed by pathology; (b) standard three-phase contrast-enhanced CT conducted less than 3 weeks prior to surgery; and (c) complete preoperative clinical data. The exclusion criteria were as follows: (a) poor image quality; (b) other malignant tumors; and (c) receipt of other treatment (e.g. chemotherapy, radiotherapy and immunotherapy) prior to the examination.

This study enrolled a total of 190 individuals. Among these, 127 patients from the Affiliated Hospital of Qingdao University were allocated to the training set, and 63 patients from the Shandong Provincial Hospital affiliated with Shandong First Medical University were allocated to the external validation set.

### Acquisition of CT images

All patients received a standard pelvic three-phase contrast-enhanced CT examination before surgery. Supplementary Table S1 lists the parameters of the CT scanning equipment. The corticomedullary-phase, nephrographic-phase, and excretory-phase images were acquired at 25, 75, and 300 s, respectively, after the thoracoabdominal aortic junction reaching a trigger threshold of 120 HU.

### Histological evaluation of PD-L1 expression status

The expression status of PD-L1 was assessed by two specialized research pathologists. Various thresholds for defining positive PD-L1 expression have been used in previous studies [22, 24–27]. In this investigation, all patients underwent transurethral resection of bladder tumor or radical cystectomy, and tissue samples were obtained after surgery. If staining for PD-L1 in immune cells represented 1% or more of the total tumor area, the tumor was considered to be PD-L1 positive [20].

### Collection and analysis of clinical and CT features

One radiologist with a decade of expertise and another with 5 years of experience assessed the CT characteristics from the images, without prior knowledge of the pathological outcomes. When they disagreed, they reached a consensus through discussion. Clinical and CT findings of the patients and their tumors were analyzed, including age, gender, CT-indicated T stage, CT-indicated N stage [27], location, shape, calcification, size, thickness, cystic necrosis, boundary, stalk, extramural infiltration, and the CT values of lesions during the cortical medullary phase (LCTV-C), nephrographic phase (LCTV-N), and excretory phase (LCTV-E). The specific standards can be found in the supplementary materials.

### Segmentation of regions of interest and extraction of radiomic features

A urological radiologist with 5 years of experience used ITK-SNAP software (version 3.8.0, http://www.itksnap.org) to manually delineate the regions of interest (ROIs) representing tumors on the CT images. All ROIs were assessed by a senior radiologist possessing a decade of experience in imaging diagnosis of BCa. All radiomic feature extraction was performed using PyRadiomics in Python. A total of 11 844 radiomic features were extracted, including shape features, first-order features, texture features, and wavelet features.

### Selection of features and construction of the machine learning model

First, a combat compensation method was used to decrease the discrepancy in barycentric radiometric characteristics [28]. Second, all features were normalized using z-scores. Third, the minimum redundancy and maximum relevance feature ranking method was used to identify 55 features with the strongest correlation and the lowest redundancy. The least absolute shrinkage and selection operator was then used to screen these features. A radiomics machine learning signature was then developed using 11 radiomics machine learning classifiers, with these including extremely randomized trees, support vector machine, random forest, k-nearest neighbor, logistic regression, naïve Bayes, light gradient boosting machine, extreme gradient boosting, gradient boosting, adaboost, and multi-layer perceptron classifiers.

### Image preprocessing and establishment of the CNN model

This signature integrated a variety of cutting-edge convolutional neural network (CNN) techniques, including channel attention mechanisms, residual blocks, mixed convolution layers, self-attention mechanisms, convolutional layers, and spatial attention mechanisms.

For the imaging, window width and level adjustments were first performed, followed by histogram equalization and normalization to enhance image contrast. The tumor slice exhibiting the largest cross-sectional area was selected for defining the ROI, and the images were subsequently cropped based on the annotated tumor boundaries. To ensure comprehensive capture of tumor margin characteristics and peripheral tissue information, a morphological dilation operation with a 6 × 6 kernel was applied to the initial ROI masks. To eliminate the influence of irrelevant information, the pixel values outside the delineation were set to zero. The images of all three phases were processed and cropped as needed, and were then combined into a single three-channel image. The stacked images were resized to 224 × 224 using bilinear interpolation [29].

To address the inherent limitations of medical imaging datasets, we implemented a systematic data augmentation strategy. The training images were rotated in 30° intervals from 0° to 330°, followed by geometric transformations, including horizontal flips, vertical flips, and their combinations.

The model was pretrained on the ImageNet1k dataset. After pretraining, it was trained on the training dataset via a cross-entropy loss function, with an imbalance parameter of 2:1 for classes 0 and 1. The Adam optimization algorithm was employed to update the parameters of the neural network. The weight decay was set to 0.001, the learning rate to 0.001, the batch size to 32, and training was conducted over more than 25 epochs. The learning rate was adjusted to 80% of its previous value every five epochs. An early stopping strategy was imposed to halt training if the loss curve converged (Supplementary Figure S1) and the calculated loss increased for two consecutive evaluations. We employed class activation maps (CAM) to observe the putative BCa regions identified by the network in order to determine PD-L1 expression status [30].

### Model interpretability with SHAP

To achieve an interpretable analysis of the model’s decision-making process, we adopted a feature attribution approach based on the Shapley value theory. SHAP is a method for interpreting the predictions of machine learning models. Specifically, we implement the SHAP framework based on expected gradients, which quantifies the importance of features in the model’s decision-making by calculating their marginal contributions [31]. For the technical implementation, GradientExplainer was used as the feature attributor. This is a theoretical extension of Integrated Gradients, Integrated Gradients is an interpretability method for interpreting the predictions of deep learning models, especially for neural network models. It is a method of Attribution that measures the extent to which input features (e.g. pixels of an image, word vectors of text, features of tabular data) contribute to the final prediction of a model. Integrated Gradients approximates the Shapley value by calculating the expected gradient for multiple samples from a reference distribution. This expected gradient-based feature imputation method maintains the theoretical properties of Shapley values (e.g. efficiency, symmetry, and linearity) while effectively handling nonlinear feature interactions in deep neural networks. With this method, we were able to generate pixel-level quantitative maps of the contribution to outcome, facilitating visualization of the decision basis of the model in the feature space, and thus providing reliable explanatory support for the clinical application of the model.

### Development of a clinical model and construction of a nomogram

We used univariate logistic regression to identify CT characteristics and clinical data associated with PD-L1 expression in BCa. Variables with a *p*-value less than 0.05 in the univariate logistic regression analysis were then included in a multivariate logistic regression analysis. Clinical features with a multi-factor p-value less than 0.05 were chosen to build the clinical model. We then selected the optimal prediction model by comparing the area under the curve (AUC) and accuracy, using the Delong test to compare receiver operating characteristics curves [28], and then used this optimum model to establish a nomogram in conjunction with the clinical model. The agreement between the classification model’s actual and anticipated outcomes was calibrated using calibration curves. Assessment of the clinical net benefit of various models was carried out using decision curve analysis.

### Statistical analysis

The clinical and CT data of all patients were analyzed using SPSS software (version 26.0, IBM). The DL signature was constructed using Python (version 3.9.7, www.python.org). Assessment of model performance and construction of the radiomics machine learning signature were based on R software (version 4.2.2, www.r-project.org). Categorical variables were analyzed using the chi-square test or Fisher’s exact test, while continuous variables were compared using the independent samples t-test or Mann–Whitney U test. P-values less than 0.05 were considered to represent a statistically significant difference.

## Results

### Clinical data and CT features of the patients

Retrospective collection of the clinical data and CT features of the patients was conducted, with 127 patients from one center being used for the training set and 63 patients from another center being used as the validation set. There were 158 male patients and 32 female patients, with ages ranging from 27 to 90 years (mean age 68.47 years). There was a significant difference in extramural infiltration between the training set and the validation set. Table 1 presents the characteristics of the patients in both sets.

**Table 1:** Clinical data and CT features of the patients

Characteristics		Training set (*n* = 127)	Validation set (*n* = 63)	*P* value
Age		67.32 ± 10.56	70.78 ± 10.68	0.419
Gender				0.714
	Male	107(84.3%)	51(81.0%)	
	Female	20(15.7%)	12(19.0%)	
T stage				0.841
	< T2	39(30.7%)	21(33.3%)	
	≥ T2	88(69.3%)	42(66.7%)	
N stage				0.054
	N0	115(90.6%)	49(77.8%)	
	N1	9(7.1%)	9(14.3%)	
	N2	3(2.4%)	5(7.9%)	
Location				0.104
	trigone	20(15.7%)	4(6.3%)	
	lateral wall	40(31.5%)	27(42.9%)	
	posterior wall	47(37.0%)	27(42.9%)	
	Roof	9(7.1%)	3(4.8%)	
	anterior wall	11(8.7%)	2(3.2%)	
Shape				0.773
	Mound-like	34(26.8%)	18(28.6%)	
	Cauliflower-like	73(57.5%)	33(52.4%)	
	Papillary	20(15.7%)	12(19.0%)	
Calcification				0.745
	Yes	22(17.3%)	9(14.3%)	
	No	105(82.7%)	54(85.7%)	
Size(cm)		9.2 ± 2.04	4.39 ± 2.00	0.183
Thickness(mm)		2.77 ± 1.32	2.71 ± 1.27	0.772
LCTV-C		68.24 ± 24.94	63.27 ± 20.92	0.842
LCTV-N		80.38 ± 21.04	79.40 ± 17.48	0.609
LCTV-E		77.74 ± 20.58	72.15 ± 21.30	0.858
Cystic necrosis				0.941
	Yes	12(9.4%)	5(7.9%)	
	No	115(90.6%)	58(92.1%)	
Boundary				0.962
	Clear	58(45.7%)	29(46.0%)	
	Vague	69(54.3%)	34(54.0%)	
Stalk				0.270
	Absent	106(83.5%)	57(90.5%)	
	Present	21(16.5%)	6(9.5%)	
Extramural infiltration				0.007
	Yes	12(9.4%)	16(25.4%)	
	No	115(90.6%)	47(74.6%)	

*T stage* CT-indicated T stage, *N stage* CT-indicated N stage, *LCTV-C* lesion CT value in the corticomedullary-phase, *LCTV-N* lesion CT value in the nephrographic-phase, *LCTV-E* lesion CT value in the excretory-phase

### Clinical model development

Table 2 summarizes the results of the univariate and multivariate logistic regression analyses. The univariate logistic regression identified two clinical features as showing a significant contribution to the prediction of PD-L1 expression status in patients with BCa. These characteristics were LCTV-N and LCTV-E, both of which had a p-value of less than 0.05. LCTV-E was also an independent risk factor for predicting PD-L1 expression status in BCa according to the multivariate logistic regression. The clinical model was therefore created using LCTV-E. The AUC values of the clinical model were 0.681 (95% confidence interval [CI]: 0.593–0.761) for the training set and 0.528 (95% CI: 0.398–0.655) for the validation set.

**Table 2:** Positive results of univariate and multivariate logistic regression of patient clinical and CT characteristics

Variable	Univariate			Multivariate		
	P	OR	95%CI	P	OR	95%CI
Age	0.677	1.007	0.974–1.042			
Gender	0.159	2.177	0.738–6.428			
T stage	0.069	2.035	0.945–4.383			
N stage	0.674	1.227	0.473–3.180			
Location	0.831	0.996	0.699–1.333			
Shape	0.122	0.640	0.364–1.127			
Size	0.837	0.982	0.824–1.169			
Thickness	0.190	1.207	0.911–1.599			
Calcification	0.086	2.550	0.875–7.430			
Cystic necrosis	0.052	0.288	0.082–1.013			
Boundary	0.080	1.922	0.926–3.989			
Stalk	0.417	0.677	0.264–1.737			
Extramural infiltration	0.654	1.333	0.379–4.686			
LCTV-C	0.298	1.008	0.993–1.023			
LCTV-N	0.016	1.024	1.004–1.044	0.449	1.009	0.986–1.033
LCTV-E	0.003	1.032	1.011–1.054	0.043	1.026	1.001–1.052

*T stage* CT-indicated T stage, *N stage* CT-indicated N stage, *CI* confidence interval, *OR* odds ratio, *LCTV-C* lesion CT value in the corticomedullary-phase, *LCTV-N* lesion CT value in the nephrographic-phase, *LCTV-E* lesion CT value in the excretory-phase

### Performance of the radiomics machine learning signature and DL signature

**Fig. 1:**
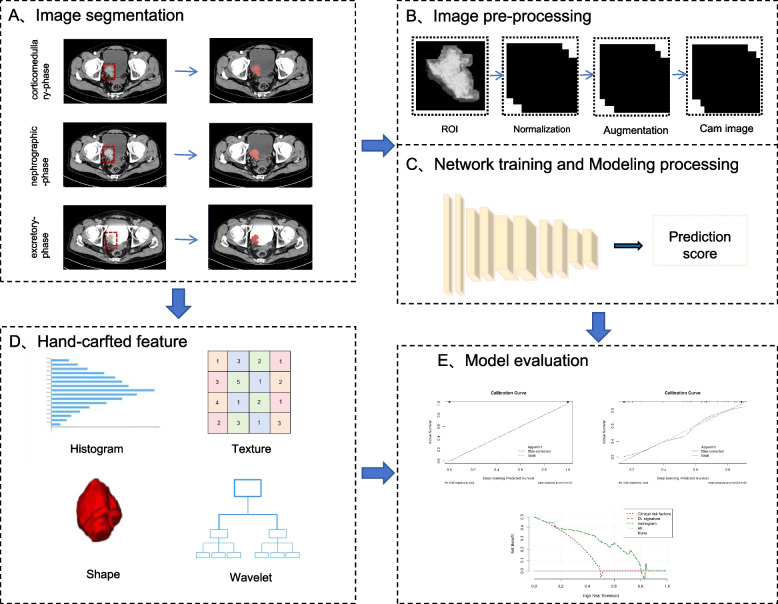
The flow chart of this study

**Fig. 2:**
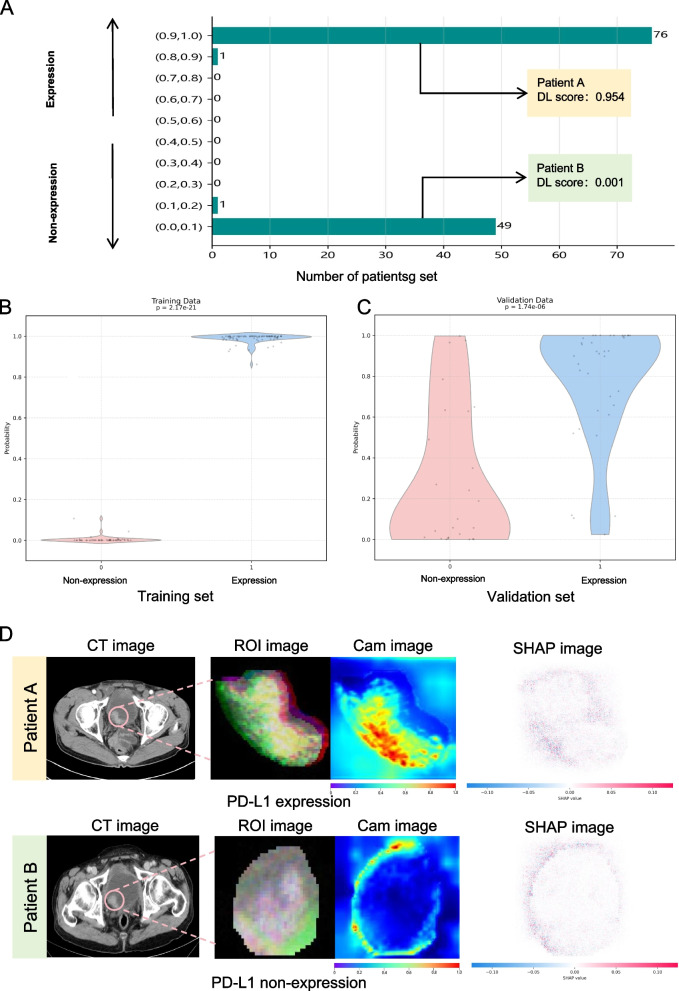
Distribution of DL scores for the training set (**A**). Relationship between DL score and PD-L1 expression status in both datasets (**B**, **C**), and the CAM and SHAP attribution map for PD-L1 expression vs non-expression (**D**)

A study flow chart is displayed in Fig. 1. The distribution of the DL scores in the training set is presented in Fig. 2A. There was a substantial difference in DL scores between the PD-L1 expression group and the non-expression group for both the training and validation sets (Fig. 2B, C). Eleven radiomics machine learning algorithms were employed. The predictive performance of the radiomics machine learning signature for predicting PD-L1 expression in BCa is described in Table S2.

#### The CAM heatmaps and model interpretability with SHAP

The activation occurring in the location of the lesion on the CAMs is one of the most important criteria for accurate prediction, and in the CNN CAMs, we can identify the highlighted activation area associated with PD-L1 expression. We found that the regions highlighted in the CAMs were mainly located in the tumor margin area near to the bladder wall (Fig. 2D). In computer vision classification tasks, where features are represented by pixels, model interpretability is helpful for determining whether pixels positively or adversely affect the category predictions. To accomplish model interpretability, we used the SHAP technique to elucidate the DL signature. This technique chiefly entails examining the gradients inside the model to attain a more profound understanding of the decision-making mechanisms. By analyzing gradients, we can identify the features that most significantly impact the model’s predictions. We present plots for PD-L1expression and PD-L1 non-expression in BCa (Fig. 2D). The original image and grayscale images that correspond to the output classes that the model predicted are included in every SHAP plot, and the contribution of the model to the output class is depicted in each grayscale image. Red pixels in these images show a positive effect, blue pixels show a negative effect, and white pixels indicate regions where the model disregarded input features. A color scale below the images delineates the spectrum from negative to positive, depicting the intensity of the SHAP values attributed to each pertinent pixel. We found that in the SHAP plots, white pixels were mainly located in the internal region of the tumor. Conversely, the predicting pixels (positive or negative effect) were mostly located in the tumor margin area, mainly the margin regions near to the bladder wall. SHAP can be used to explain to physicians how deep learning features of bladder cancer affect overall predictive outcomes.

#### Construction of the nomogram and evaluation of the different models

**Fig. 3:**
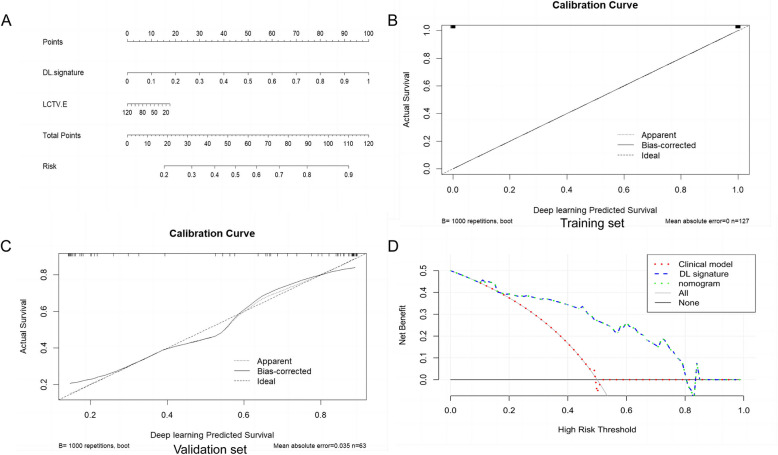
The nomogram including clinical risk factors and the DL signature (**A**). Calibration curves of the DL signature for the training and validation sets (**B**, **C**). Analysis of decision curves for various models (**D**)

Compared with the radiomics analysis, the DL signature had better prediction performance. The nomogram was constructed by integrating the DL signature with the clinically independent risk factors (Fig. 3A). Table 3 illustrates the predictive performance of the DL signature, nomogram, and clinical model. We used AUC, sensitivity, specificity, and accuracy to judge the performance of the three models. The discrimination of the DL signatures was found to be optimal. The external validation set revealed that the DL signatures had the highest AUC of 0.857 (95% CI: 0.745–0.932). The Delong test indicated that the diagnostic efficacies of the nomogram and DL signature were much superior to that of the clinical model for both data sets (both P < 0.05). Calibration plots for predicting PD-L1 expression status (Fig. 3B, C) showed that the DL signature was well calibrated for both datasets. Decision curve (Fig. 3D) analysis showed that the nomogram and the DL signature had greater net clinical benefit.

**Table 3:** DL signature, clinical model, and nomogram predictive performance

	Training set			Validation set		
	DL signature	Clinical model	Nomogram	DL signature	Clinical model	Nomogram
AUC(95%CI)	1(0.971–1.000)	0.681(0.593–0.761)	1(0.971–1.000)	0.857(0.745–0.932)	0.528(0.398–0.655)	0.857(0.745–0.932)
ACC	1	0.677	1	0.825	0.603	0.825
SEN	1	0.610	1	0.892	0.541	0.892
SPE	1	0.780	1	0.731	0.684	0.731
*P*-value	< 0.001*	Reference	< 0.001*	0.001*	Reference	0.001*

*AUC* area under the curve, *ACC* accuracy, *CI* confidence interval, *SPE* specificity, *SEN* sensitivity

^*^ In comparison with the Clinical model

## Discussion

In this study, we created and assessed a 3D-CNN framework based on three-phase contrast-enhanced CT images for predicting the preoperative PD-L1 expression status of BCa. The DL signature performed well on the external validation set (AUC: 0.857; 95%CI: 0.745–0.932). The results demonstrate that the DL signature for predicting PD-L1 expression in BCa exhibited optimal predictive ability, high accuracy, and superior clinical benefits, underscoring its stability and universality. The signature may be an effective tool for assisting preoperative clinical treatment decisions.

In the study by Cao et al. [20], univariate analysis failed to find any clinically significant features that predicted the expression status of PD-L1 in BCa (*P* < 0.05). In this study, despite gathering nearly all accessible clinical and CT information of the patients, the clinical model exhibited subpar performance on both datasets. Moreover, the addition of clinical variables did not improve the predictive performance of the DL signature. This result indicates that the clinical model’s ability to predict PD-L1 expression status in BCa is limited.

Radiomics provides non-invasive methodologies for discerning tumor biological behavior through the extraction and analysis of high-throughput quantitative data obtained from medical imaging. Numerous research studies have examined the connection between radiomics signatures and PD-L1 expression in various types of solid tumors in order to support immunotherapy treatment decisions [32–34]. We used 11 radiomics machine learning algorithms for predicting PD-L1 expression status in BCa, and among these, the light gradient boosting machine model yielded the highest performance, achieving an AUC of 0.692 and accuracy of 0.714. The predictive performance of our machine learning model is on the same level as the results of Cao et al. [20]. The prediction capabilities of the radiomics machine learning signature fall short of the requirement for clinical applications.

DL algorithms, which use specific functions in their hidden layers to describe in-depth intratumor heterogeneity [35], have illustrated the significance of the DL signature derived from CT imaging in the diagnosis and therapy of BCa [22–24]. In the current research, we employed a CNN for supervised end-to-end training, which significantly streamlined the training procedure and achieved the best predictive performance. The dataset exhibited heterogeneity in image acquisition parameters, mirroring real clinical scenarios, and indicating that DL methods may possess adequate robustness and generalizability for practical applications. The CNN CAMs contained important regions related to PD-L1 expression status. The highlighted areas in CAMs and SHAP plots are mainly located in the tumor edge regions close to the bladder wall. This might be related to the fact that the tumor margin is a key area for tumor invasion and immune response [36, 37], and PD-L1 promotes immune escape by inhibiting T lymphocyte activity [38]. To overcome the “black box” problem of DL models, we used SHAP technology to generate pixel-level quantitative maps to visualize the contribution to the decision of the model in the feature space thus the application of Shapley additive explanation technology can assist physicians in comprehending the influence of DL signatures on model prediction outcomes.

PD-L1 are predictors of immune response to treatment [8], tissue sampling is a commonly used method but is not always feasible and is associated with high costs, limiting the application of invasive biopsy-based molecular assays. In this regard, deep learning-based approaches can be used as an adjunct to tissue-based biomarkers.

Our study has several limitations. First, the performance analysis is retrospective, with a limited sample size and the potential for introducing selection bias; therefore, extensive prospective studies are necessary for validation. Second, we did not investigate the fluctuations and variable expression of PD-L1 throughout the progression of malignant tumors or following treatment. Third, the therapeutic response after immunotherapy was not evaluated. Consequently, we plan to develop models to predict immunotherapy responses in the future. Fourth, the potential limitations or challenges associated with using SHAP, (1) For high-resolution images, iterative calculation of SHAP values consumes a lot of computational resources, which may cause a bottleneck in real-time clinical decision scenarios. (2) SHAP was sensitive to noise. In medical imaging scenarios, noise and artifacts (such as motion artifacts, metal artifacts, etc.) caused by differences in equipment parameters and different image acquisition conditions may lead to unstable or even incorrect interpretation of feature importance by SHAP.

## Conclusion

In this study, we designed an easily-accessible noninvasive and efficient DL signature to predict PD-L1 expression in BCa. The SHAP framework aids physicians and patients in comprehending the internal prediction process, thereby enhancing the credibility of the DL signature. Interpretable DL signature can enhance patient trust, optimize personalized treatment, improve clinicians’ decision-making ability. We recommend using the DL signature for assistance in clinical trial design and for bridging the gap between precision medicine and radiography.

## Supplementary Information

Supplementary Material 1: Table S1. Parameters used for CT imaging at the two medical centers. Table S2. The performance of the different radiomics machine learning signatures. Figure S1. Convergence of the loss curve. Supplementary materials.

## Data Availability

No datasets were generated or analysed during the current study.

## Abbreviations

AUC: Area under the curve
BCa: Bladder cancer
CT: Computed tomography
CNN: Convolutional neural networks
CAM: Class activation maps
DL: Deep learning
LCTV-C: Lesion CT value in corticomedullary-phase
LCTV-N: Lesion CT value in nephrographic-phase
LCTV-E: Lesion CT value in excretory-phase
PD-L1: Programmed cell death ligand 1
ROIs: Regions of interest
SHAP: Shapley additive explanation
