# Supplementary material for: A CT-based interpretable deep learning signature for predicting PD-L1 expression in bladder cancer: a two-center study
Source: Cancer Imaging. 2025 Mar 10;25:27. doi: 10.1186/s40644-025-00849-1 (PMC11892212; doi:10.1186/s40644-025-00849-1)
Supplement: Supplementary file 1 — Supplementary Material 1: Table S1. Parameters used for CT imaging at the two medical centers. Table S2. The performance of the different radiomics machine learning signatures. Figure S1. Convergence of the loss curve. Supplementary materials. [file 40644_2025_849_MOESM1_ESM.docx]

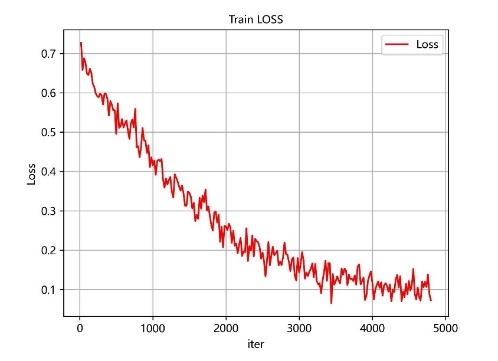


Figure S1 Convergence of the loss curve

**Table S1** Parameters used for CT imaging at the two medical centers.

| Parameters | Affiliated Hospital of Qingdao University | Shandong Provincial Hospital Affiliated to Shandong First Medical University |
| --- | --- | --- |
| CT version | Aquilion ONE 640, TOSHIBA; Discovery 750, GE Healthcare; Somatom Sensation Cardiac 64, Siemens Healthcare | Aquilion ONE, TOSHIBA; Discovery 750, GE Healthcare; Somatom Definition Flash, Siemens Healthcare |
| Tube voltage | 120kV | 120kV |
| Tube current | automatic tube current modulation | automatic tube current modulation |
| Detector collimation | 64×0.6mm  or 64×0.625mm | 64×0.6mm  or 64×0.625mm |
| Image matrix | 512×512 | 512×512 |
| Pitch | 0.9 | 0.9 |
| Contrast material | Ultravist 370  or Omnipaque 350 | Omnipaque 350 |
| Contrast medium dose | 80ml | 80~90ml |
| Injection rate | 3.0ml/s | 3.0ml/s |
| Reconstruction slice thickness | 5mm | 5mm |

Note: kV = kilovolt, mm = millimeter

**Table S2** The performance of the different radiomics machine learning signatures

|  | Training set | | Validation set | |
| --- | --- | --- | --- | --- |
| classifier | AUC(95%CI) | ACC | AUC(95%CI) | ACC |
| Logistic regression | 0.881(0.825-0.938) | 0.811 | 0.646(0.503-0.789) | 0.667 |
| Naivebayes | 0.806(0.727-0.886) | 0.772 | 0.472(0.321-0.623) | 0.571 |
| Support vector machine | 0.917(0.867-0.967) | 0.843 | 0.652(0.509-0.794) | 0.667 |
| K nearest neighbor | 0.895(0.845-0.946) | 0.630 | 0.593(0.453-0.733) | 0.460 |
| Randomforest | 0.997(0.991-1.000) | 0.945 | 0.587(0.441-0.733) | 0.429 |
| Extremely randomized trees | 1.000(1.000-1.000) | 0.394 | 0.611(0.470-0.751) | 0.540 |
| Extreme gradient boosting | 1.000(1.000-1.000) | 0.992 | 0.568(0.422-0.714) | 0.556 |
| Lightgradient Boosting machine | 0.892(0.837-0.946) | 0.827 | 0.692(0.552-0.832) | 0.714 |
| Gradientboosting | 0.948(0.912-0.983) | 0.866 | 0.619(0.476-0.763) | 0.635 |
| Adaboost | 0.941(0.905-0.977) | 0.835 | 0.480(0.332-0.628) | 0.587 |
| Multi-layer Perceptron | 0.885(0.829-0.941) | 0.795 | 0.579(0.431-0.727) | 0.619 |

Note: AUC, area under the curve; ACC, accuracy; CI, confidence interval

Supplementary materials

Regarding N-stage: Lymph nodes with a short diameter greater than 1 cm are considered to have lymph node metastasis.

N-----regional lymph nodes

Nx regional lymph nodes could not be evaluated

No No regional lymph node metastasis was observed

N1 Lymph node metastasis in single region of true pelvis (perivesical, obturator,

internal/external iliac, anterior sacral lymph node metastasis)

N2 Multiple regional lymph node metastases in true pelvis (perivesical, obturator,

internal/external iliac, anterior sacral lymph node metastases)

N3 common iliac artery lymph node metastasis.

Regarding the Cystic necrosis : Cystic necrosis can be manifested as low density shadow in the mass on CT, and CT value is the water density value. When the enhanced CT scan was performed, there was no enhancement in the necrotic area.

Regarding the measurement of CT values: The key to conducting CT value measurement at each stage lies in the selection of the region of interest (ROI), which should be located at the center of the maximum layer of the tumor lesion in the CT image (2D image), and the average value of three measurement values should be taken.
